# Editorial: Novel approaches to study metals in molecular biology

**DOI:** 10.3389/fmolb.2023.1167896

**Published:** 2023-02-27

**Authors:** Teresita Padilla-Benavides, Katherine E. Vest, Alma D. Campos-Parra, Daniel Raimunda

**Affiliations:** ^1^ Molecular Biology and Biochemistry Department, Wesleyan University, Middletown, CT, United States; ^2^ Department of Molecular and Cellular Biosciences, University of Cincinnati College of Medicine, Cincinnati, OH, United States; ^3^ Instituto de Salud Pública, Universidad Veracruzana, Xalapa, Veracruz, Mexico; ^4^ Instituto de Investigación Médica Mercedes y Martín Ferreyra-INIMEC-CONICET, UNC, Córdoba, Argentina

**Keywords:** transition metals, homeostasis, metal transporters, metallochaperones, metal toxicity

## Introduction

Trace elements are essential nutrients for multiple biological reactions. Metals like copper (Cu), zinc (Zn), iron (Fe), and manganese (Mn) are catalytic or structural cofactors of enzymes involved in various metabolic pathways. Trace elements can be toxic at elevated concentrations, especially those capable of cycling between oxidation states, and imbalance of metals leads to deleterious biological effects. Therefore, cellular uptake and distribution of metals is strictly controlled. Organisms have evolved regulatory systems that collectively orchestrate metal homeostasis. These include membrane barriers, transporters, and soluble sensors and chaperones. Although several mechanisms of metal homeostasis have been identified in prokaryotes and eukaryotes, several questions remain unresolved, including the impact of altered metal homeostasis on human health and development. This Research Topic emphasized novel interdisciplinary research and biological systems toward understanding the fundamental mechanisms of metal homeostasis regulation. This special edition contains six manuscripts focused on the roles of trace elements in various biological models and are summarized below.

## Metals and microbes: Infection, immunity, and environmental remediation

Interactions between metal homeostasis and the immune system have received special attention since the discovery of nutritional immunity in response to bacterial infection. However, little is known about the importance of metals in the host response to viral infection. Vasquez-Procopio et al. correlated inflammatory markers, severity of disease and metal levels in the serum of COVID-19 infected individuals during the first trimester of pregnancy. The study recruited 197 pregnant women of which 163 were positive for COVID-19, including 76 asymptomatic, 64 mild and 23 severe clinical manifestations. Severe manifestations correlated with increased Mg, Cu, and Ca levels and decreased Fe, Zn, and Na levels in serum. The inflammatory markers IL-6, TNF-α, IL-8, IL-1α, the anti-inflammatory cytokine IL-4 and the chemokine IP-10 were stimulated in severe cases of COVID-19 during pregnancy. Negative correlations between Fe/Mg and Zn/IL-6, and a positive correlation between Cu/IP-10 were observed in patients presenting with severe disease. Alteration in serum metals and inflammatory markers in pregnant women infected with COVID-19 may contribute to the severity of the disease.

Although bacteria use Cu as a cofactor, they are particularly sensitive to its toxicity and Cu acts as an antibacterial component of host immunity. Peralta et al. demonstrated a dual role for the siderophore enterobactin in preventing or promoting Cu toxicity in Enterobacteriaciae. Many enterobacteria, including *Escherichia coli*, produce enterobactin, which can reduce Cu^2+^, enhancing Cu toxicity. The authors used a mutant strain of *E. coli* deficient of enterobactin that is sensitive to Cu toxicity. Low concentrations of enterobactin had a protective effect by reducing intracellular ROS upon hydrolysis. High-levels of enterobactin resulted in bacterial death in the presence of Cu, likely due to the reduction of Cu^2+^. Thus, enterobactin has a bimodal effect-protective or deleterious-depending on its concentration.

Divalent Mn, commonly found in groundwater, negatively impacts water quality, disinfection and distribution, and in excess can harm human health. Certain bacterial species can form biofilms and oxidize Mn^2+^, providing an efficient way to improve traditional water sand filtration methods. However, this process is less efficient during winter months as low temperatures alter biofilm formation. Species like *Pseudomonas* sp. MOB-449 (MOB-449) form biofilms and oxidize Mn^2+^ even at lower temperatures. Ciancio Casalini et al., described an enhanced biofilm formation in MOB-449 by Mn^2+^ supplementation at low temperatures. Transcriptional analyses revealed that terminal oxidases and genes related to cytochrome c biogenesis are induced by these conditions. The authors propose that in MOB-449 Mn^2+^ oxidation is activated at low temperatures as a potential mechanism to produce energy, suggesting an additional physiological role for this ion.

## Metals in mammalian cells: Regulation at every level

Mitochondrial Cu is required for activity of cytochrome c oxidase (COX) and the intermembrane space pool of the Cu, Zn superoxide dismutase. The mammalian homolog of the yeast mitochondrial Cu transporter Pic2, SLC25A3, is a phosphate carrier that has been previously demonstrated to transport Cu. McCann et al., using confocal microscopy analyses, immunoprecipitation and *in vitro* Cu^+^-transfer experiments showed that SLC25A3 may contribute to Cu delivery into COX favoring the growth and differentiation of primary myoblasts. Deletion of *SLC25A3* delayed proliferation and differentiation of primary myoblasts, which was rescued by Cu supplementation. The promoter region of *SLC25A3* gene is a target of the metal-responsive transcription factor MTF1 in an interaction enhanced by Cu. The model is in agreement with the previously identified role of MTF1 promoting myogenesis and SLC25A3 delivering Cu to COX during myotube formation.

The Lutsenko laboratory showed that the heterogeneous nuclear ribonucleoprotein hnRNPA2/B1 regulates Cu homeostasis by modulating the abundance of ATP7A *via* the 3′ untranslated region (UTR) in the *ATP7A* mRNA in HeLa and SH-SY5Y neuroblastoma cells. hnRNPA2/B1 deficiency resulted in increased levels of the *ATP7A* transcript and protein which correlated with a decrease in cellular Cu. Knockdown of the isoforms B1 and B1b of hnRNPA2/B1 is sufficient to elevate *ATP7A* levels. Conversely, overexpression of the hnRNPA2 or hnRNPB1 isoforms reduced the levels of the *ATP7A* transcript. The authors concluded that hnRNPA2/B1 is a negative regulator of *ATP7A* abundance and therefore intracellular Cu levels.

Philpott et al. presented an enticing review discussing the contributions of the poly C-binding RNA proteins (PCBP1, PCBP2) to Fe trafficking in mammals. Several enzymes use Fe cofactors, as Fe/S clusters, or mono- or di-nuclear Fe centers. Cytosolic Fe chaperones are essential for supplying the metal cofactors to their targets in mammalian cells. Among these chaperones, PCBP1 delivers Fe to ferritin, as well as multiple cytosolic Fe enzymes. PCBP2 shuttles Fe from the DMT1-dependent import pathway to ferroportin-dependent export route. This review presents a detailed description of classic and novel approaches that revealed the diverse roles and contributions of PCBP1 and PCBP2 in cytosolic Fe management.

## Summary and concluding remarks

Essential roles for metals continue to emerge and research is moving forward taking advantage of novel powerful methodologies and model systems. It is undeniable that our understanding of these essential ions is still on the rise, and efforts should continue to uncover novel roles and mechanisms associated with metals in organisms.

